# Epidemiological Investigation of Infectious Diseases at the Domestic–Synanthropic–Wild Animal Interface Reveals Threats to Endangered Species Reintroduction in AlUla, Saudi Arabia

**DOI:** 10.3390/vetsci12090836

**Published:** 2025-08-30

**Authors:** Sulaiman F. Aljasir, Abdelmaged A. Draz, Bilal Aslam, Abdullah S. M. Aljohani, Madeh Sadan, Nawaf Al-Johani, Ayman Elbehiry, Waleed Al Abdulmonem, Musaad Aldubaib, Basheer Aldurubi, Abdulhakim M. Alyahya, Abdulmalik Alduhami, Abdulaziz Aljaralh, Moh A. Alkhamis, Jeffrey C. Chandler, Bledar Bisha, Osama B. Mohammed

**Affiliations:** 1 Department of Veterinary Preventive Medicine, College of Veterinary Medicine, Qassim University, Buraydah 52571, Saudi Arabia; draz@qu.edu.sa (A.A.D.); b.aslam@qu.edu.sa (B.A.); basherdroubi@gmail.com (B.A.); abd.alyahya@qu.edu.sa (A.M.A.); abdualmalik-16@hotmail.com (A.A.); abdulaziz.aljaralh@gmail.com (A.A.); 2 Department of Medical Biosciences, College of Veterinary Medicine, Qassim University, Buraydah 52571, Saudi Arabia; jhny@qu.edu.sa; 3 Department of Clinical Sciences, College of Veterinary Medicine, Qassim University, Buraydah 52571, Saudi Arabia; m.sadan@qu.edu.sa; 4 The Royal Commission for AlUla, Riyadh 12512, Saudi Arabia; n.aljohani@rcu.gov.sa; 5 Department of Public Health, College of Applied Medical Sciences, Qassim University, Buraydah 52571, Saudi Arabia; ar.elbehiry@qu.edu.sa; 6 Department of Pathology, College of Medicine, Qassim University, Buraydah 52571, Saudi Arabia; dr.waleedmonem@qu.edu.sa; 7 Department of Pathology and Laboratory Diagnosis, College of Veterinary Medicine, Qassim University, Buraydah 52571, Saudi Arabia; drmusaad@qu.edu.sa; 8 Department of Epidemiology and Biostatistics, College of Public Health, Health Sciences Centre, Kuwait University, Kuwait City P.O. Box 5969, Kuwait; m.alkhamis@ku.edu.kw; 9 Wildlife Disease Diagnostic Laboratory, Wildlife Services, Animal and Plant Health Inspection Service, United States Department of Agriculture, Fort Collins, CO 80521, USA; jeffrey.c.chandler@usda.gov; 10 Department of Animal Science, College of Agriculture and Natural Resources, University of Wyoming, Laramie, WY 82071, USA; bbisha@uwyo.edu; 11 Department of Zoology, College of Science, King Saud University, Riyadh 12372, Saudi Arabia; omohammed@ksu.edu.sa

**Keywords:** AlUla, domestic–wildlife interface, infectious diseases, wildlife, endangered species conservation, disease prioritization, wildlife disease management, synanthropic animals, wildlife reintroduction

## Abstract

AlUla, a unique conservation and tourism hub in Saudi Arabia, is undergoing extensive biodiversity restoration efforts, including the reintroduction of threatened wild species. However, interactions among wildlife, domestic, and synanthropic animals in these reserves raise significant concerns about disease transmission to reintroduced species. This study aimed to assess disease risks at the domestic–synanthropic–wildlife interface and identify infectious diseases posing the greatest threat to reintroduced species. A multi-phased prioritization system was developed to guide monitoring based on transmissibility to protected wildlife, susceptibility of reintroduced species, reservoir hosts, vector-borne potential, likelihood of occurrence, and disease severity. A comprehensive expert review identified 61 diseases important to the reintroduced wildlife. From this, 11 priority pathogens were selected for monitoring. A total of 7760 samples were collected from 1367 domestic and synanthropic animals and were analyzed using Real-Time PCR and/or ELISA. All priority pathogens, or prior exposure to these pathogens, were detected. Disease presence was affected by factors such as species, location, health status, and grazing habits. Taken together, these findings underscore the need for robust preventive measures to mitigate disease transmission risks and ensure the sustainability of AlUla’s conservation initiatives. This study also offers a model approach to support reintroduction programs and guide future conservation efforts.

## Simple Summary

AlUla, a historic region in Saudi Arabia, is undergoing extensive restoration of its natural biodiversity by reintroducing endangered wildlife species. However, these efforts face growing challenges, as domestic animals (camels, sheep, goats, cattle) and feral animals (donkeys, dogs, cats, rodents) share space and resources, increasing the risk of disease spread to wildlife. This study aims to investigate the prevalence of some infectious diseases and assess which of them could be circulating among domestic, feral, and wild animals in AlUla and Khaybar. Expert review identified 61 diseases potentially present in the area. A scoring system was used to prioritize 11 of these diseases for further investigation. Blood, swabs, and other samples were collected from animals and tested using laboratory techniques to detect different disease agents. Evidence of exposure or detection of 11 of these pathogens were observed in AlUla domestic and feral animals. These findings suggest that endangered wildlife reintroduced or intended for reintroduction may face increased risk of acquiring infectious diseases from domestic and feral animals. This study proactively assessed pathogen threats in advance of reintroduction, highlighting the need for targeted disease monitoring, vaccination, and other control measures to protect animal health and ensure the success of conservation programs in AlUla.

## 1. Introduction

The animal–environmental interface acts as a critical nexus for disease transmission, holding significant epidemiological implications for human and animal health, as well as nature conservation [1]. This interface often facilitates the spillover of infectious agents, primarily driven by animal farming, shared habitats, resource competition, and anthropogenic activities such as deforestation and hunting. Such interactions can increase disease incidence, threaten wildlife populations, and undermine agricultural and livestock productivity [2]. Understanding the dynamics and epidemiological landscape at this interface are essential for developing comprehensive preventive and management strategies to address infectious diseases and ensure our success with these effective nature conservation programs [3].

AlUla County is currently undergoing a transformation into a prominent center for tourism and nature conservation, aligning with Saudi Vision 2030, which aims to significantly enhance regional tourism and protection of associated natural habitats. The unique structure of AlUla, characterized by multiple small towns interspersed throughout areas of historically minimal anthropogenic disturbance, offers an ecologically favorable landscape for the reintroduction of keystone wildlife species [4,5]. The region supports diverse populations of domestic, synanthropic, and native wild animals. Synanthropic species such as feral donkeys (*Equus asinus*), cats (*Felis catus*), dogs (*Canis lupus familiaris*), and rodents (*Meriones libycus* and *Jaculus jaculus*) have become increasingly prevalent in anthropogenic landscapes. Domestic animals, including camels (*Camelus dromedarius*), cattle (*Bos taurus*), sheep (*Ovis aries*), and goats (*Capra hircus*), remain integral to local livelihoods. Recent assessments estimated the density of feral donkeys at approximately 1.03 animals per km^2^ [6], while camel populations exceed 10,000 and small ruminants, including sheep and goats, surpass 30,000 across AlUla County (unpublished report).

Interactions between domestic and synanthropic animals with native and reintroduced wild species, including the highly endangered Arabian oryx, Nubian ibex, as well as the sand and mountain gazelles, may pose risks for the spillover of infectious diseases, with potential conservation, economic, and animal health consequences [7]. Recent conservation initiatives in AlUla have established protected reserves in areas previously exposed to limited human activities, and native wildlife species have since been reintroduced into parts of their ancestral habitats. Consequently, understanding and identifying the risks posed by domestic and synanthropic animals to both reintroduced and existing wild populations warrants thorough epidemiological investigation to guide effective disease prevention strategies and ensure the long-term success of conservation efforts [5].

The primary objective of this study was to characterize the presence and distribution of pathogens in AlUla and Khaybar to better support the reintroduction of native wildlife and mitigate the potential impact of disease transmission among wild, domestic, and synanthropic animals. To achieve this, an epidemiological investigation was conducted to report the prevalence of contagious animal diseases that may be transmitted from domestic and synanthropic animals to the highly threatened wild species, including the Arabian oryx, Nubian ibex, as well as sand and mountain gazelles. In addition, the study aimed to provide responsible authorities with targeted, evidence-based recommendations for the prevention and control of disease spread in AlUla region.

## 2. Materials and Methods

### 2.1. Study Settings

**Figure 1:**
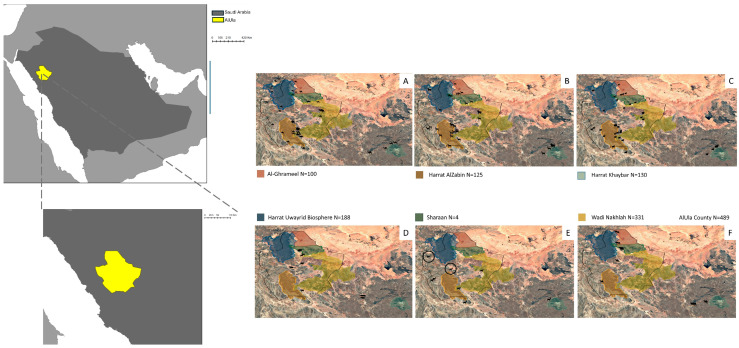
Geographic location of AlUla County within Saudi Arabia. Sampling locations, each sample location corresponds to an area of ~2.5 km^2^ (**A**). The average number of camels sampled per location is 5, with a range from 1 to 20. (**B**) The average number of goats sampled per location is 7, with a range from 1 to 17. (**C**) The average number of sheep sampled per location is 6, with a range from 1 to 20. (**D**) The average number of cattle sampled per location is 5, with a range from 2 to 10. (**E**) The number of donkeys sampled per location is 2, except in the circled locations where the number of sampled animals was 51. (**F**) Each sample location corresponds to an area of ~3 km^2^; the average number of Rodents sampled per location is 10, with a range from 1 to 25. Details of the sample size for each species within each reserve are provided in Supplementary Table S3.

The targeted conservation regions or protected areas included Al-Ghrameel, Harrat Uwayrid Biosphere, Sharaan, Harrat AlZabin, Wadi Nakhlah, and Harrat Khaybar, ranging in size from 600 to 4680 km^2^. All are located within AlUla County (Figure 1), except for Harrat Khaybar, which lies just beyond its southern extent, but remains ecologically connected through natural wildlife movement. Of these reserves, Sharaan (1550 km^2^) is the only fenced area. AlUla County experiences hot summers from May to September, with temperatures frequently exceeding 35 °C, peaking in August and September at average daily highs of 38 °C and lows between 20 and 22 °C. Winters (from December to February) are cooler, with daily highs generally below 24 °C; the coldest months, January and February, have average lows of 4–5 °C and highs of 21–22 °C. The International Union for Conservation of Nature (IUCN) has documented 32 mammalian species within the region.

### 2.2. Expert Review and Disease Prioritization

A comprehensive expert review was conducted using multiple information sources to identify and evaluate diseases prevalent in the target region. These sources included published literature, World Organization for Animal Health (WOAH), reports from private and governmental sectors, along with field data collected through on-site visits and interviews with veterinarians, animal owners, and breeders. To prioritize diseases effectively, a multi-faceted system was developed to optimize our efforts toward diseases that might pose the greatest risk to reintroducing wild animals [8]. The criteria determined to be most important included the following factors: transmissibility to wildlife, potential susceptibility of reintroduced species (Arabian oryx, Nubian ibex, sand and mountain gazelles), reservoir host characteristics, disease etiology, likelihood of occurrence in the target region (very likely, likely, possible, etc.), disease severity (catastrophic, critical, moderate, or minor), and expert opinion. The scoring scheme assigned weighted values to each criterion, with thresholds established to classify diseases as highly or moderately important. Only diseases categorized as highly important were selected for on-field screening. A detailed description of the scoring methodology, including scoring definitions and individual weightings, is provided in (Supplementary Table S1).

### 2.3. Sample Collection

Sample size calculation was performed using probability sampling to ensure representative coverage. Animals were categorized into domestic {camels (*Camelus dromedarius*), cattle (*Bos taurus*), sheep (*Ovis aries*) and goats (*Capra hircus*)} and synanthropic {donkeys (*Equus acinus*), dogs (*Canis falmiaris*), cats (*Felis catus domesticus*), and rodents including gerbils (*Meriones libycus*) and Jerboa (*Jaculus jaculus*) }. Each species were treated as a stratum, and sample sizes were calculated separately for each species using the formula *S* = *Z*^2^ X *P* X (1 − *P*)/*M*^2^, where *S* is the sample size, *Z* is the Z-score corresponding to the 95% confidence level, *P* is the expected prevalence (set at 0.5 for all diseases), and *M* is the margin of error (set at 0.1) [9].

A total of 7760 samples were collected from 1367 randomly selected animals using a stratified random sampling method within and around AlUla protected reserves from October 2023 to May 2024. Each target area was divided into 4–6 units reflecting the reserve boundaries. Due to the dynamic nature of animal movements and the non-fixed locations of herds, the sample size for each unit was determined based on the population density at the time of sampling. The sampling distribution for each animal across reserves is shown in Figure 1. Feral cats and dogs were captured by AlUla Animal Welfare Center using baited traps. However, detailed location data for these captures were not available.

Blood samples were collected via jugular venipuncture using EDTA tubes (Jiangsu Kangbao, China), while unclotted blood samples were collected using Gel Activator tubes (Alweqaya, Saudi Arabia) under manual restraint. EDTA tubes underwent immediate gentle agitation, whereas serum samples were allowed to clot at room temperature for ~2 h. After clot separation, samples were centrifuged at 2500× *g* for 10 min, then the serum was pipetted and stored in cryogenic tubes (Thermo Fisher Scientific, Waltham, MA, USA) at −20 °C for subsequent analyses.

As for animal restraint, camels were restrained in a sitting position with both forelimbs and hind limbs tied securely before sampling. Sheep and goats were controlled by approaching from the blind spot and holding firmly beneath the chin during swab collection. Cattle were managed using nose tongs or a manual nose twitch, with tail restraint applied when needed. Donkeys required manual restraint by a team of four trained staff members who held the animal securely to allow safe collection of samples.

A minimum of 10 g of fresh feces was aseptically collected directly from the animal’s rectum using sterile disposable plastic spoons. Urine and milk samples were also collected in sterile universal containers (Last Made, Saudi Arabia). Vaginal, oral, nasal, and rectal swabs were obtained using GenoTube Livestock swabs (Thermo Fisher Scientific), as well as viral and bacterial swabs (SAMCO, Al Jubail, Saudi Arabia). In addition, post-mortem samples were collected in sterile universal containers (Last Made).

Commercial traps (KANA, Saudi Arabia) baited with bread, peanuts, or oats were used to trap rodents. Traps were set at sunset under a tree or in a well-secured location and were checked before sunrise to collect the captured animals. Empty traps were reset for the following day. Rodents were sacrificed by decapitation using a guillotine, and blood samples were collected either from the retro-orbital plexus or directly from the heart. All rodents were anesthetized with an individual weight-dependent IP dose of ketamine (10%; Dopalen, Ceva Sau’de Animal Ltd.a, Paulı’nia–SP, Brazil) 10% (1 mg/kg BW) and xylazine (Xylased Bioveta, a.s, Ivanovice na Hane’, Czech Republic) and 2% (0.25 mg/kg BW). Intestines were also collected and placed in sterile containers. All samples were transported to the One Health Laboratory at Qassim University under refrigerated conditions (4 °C), and were stored at −80 °C until further analyses.

### 2.4. Molecular and Serological Analyses

Samples were subjected to respective DNA extraction kits according to the manufacturer’s protocol. Different kits were used for the extraction of DNA, including Thermo Scientific GeneJET™ Genomic DNA Purification Kit for blood samples, PureLink™ Microbiome DNA Purification Kit for fecal samples, GeneJET™ Viral DNA and RNA Purification Kit, Thermo Scientific GeneJET™ Genomic DNA Purification Kit for Gram-Negative bacteria, and Thermo Scientific GeneJET™ Genomic DNA Purification Kit for Gram-Positive bacteria. Extracted DNA was quantified using a Nanodrop spectrophotometer (NanoDrop One/Onec/™, ThermoFisher Scientific).

For quantitative real-time PCR (qPCR) assays, several kits and reagents were used, each with specific components, volumes, thermal profiles, and primers (Table 1). PCR kits used in this study included the Solifast Probe qPCR Mix with UNG 5X kit (Solis BioDyne, Tartu, Estonia), Taq Path BactoPure Microbial Detection Kit with Rox (Applied Biosystems, Foster City, CA, USA), Ag-Path-ID™ One-Step RT-PCR Kit (Life Technologies, Waltham, MA, USA), and DreamTaq PCR Master Mix (2X) (Life Technologies). All qPCR assays were conducted using the QuantStudio™ 5 Real-Time PCR System (Applied Biosystems). Reverse transcription for foot and mouth disease (FMD) and peste des petits ruminants (PPR) was conducted at 45 °C for 10 min. PCR mixtures (50 µL) contained 25 µL DreamTaq Green PCR Master Mix, 1 µL of both forward and reverse primers (10 µM), µL 0.5 TaqMan^®^ Probe (10 μM), 12.5 µL Template DNA, and 10 µL nuclease-free water. PCR products were visualized through 1.5% agarose gel electrophoresis and under ultraviolet illumination using a gel documentation system.

Enzyme-Linked Immunosorbent Assay (ELISA) protocols were optimized according to each target disease by modifying antibody concentrations, incubation conditions, and detection methods. The ELISA kits used in the study included PrioCHECK™ *Brucella* Ab 2.0 Strip Kit (ThermoFisher Scientific), Multiscreen AgELISA Enterotoxemia/sandwich, double wells, Bovine *Pasteurella multocida* IgG Antibody ELISA Kit, *Mycoplasma capricolum* subsp. *capripneumoniae* (Mccp) Antibody Test Kit (IDEXX CCPP, Westbrook, ME, USA), PrioCHECK™ Ruminant Q Fever Ab Plate Kit (ThermoFisher Scientific), PrioCHECK™ FMDV NS Ab Plate Kit, the IDEXX Chlamydiosis Total Ab Test, Abbexa Cow Theileria Antibody ELISA Kit, ID Screen^®^ PPR Competition ELISA kit, and IDEXX and ID Vet (Innovative Di-agnostics, Grabels, France) kits for tuberculosis and toxoplasmosis.

### 2.5. Statistical Analysis

Data analysis was performed using STATA software (version 16.0). The mean age and its standard deviation were estimated for each sampled animal species, and categorical variables were summarized into frequencies and relative frequencies. Statistically significant relationships between the disease status of the animal and selected risk factors, including species, husbandry practices, and sex, were investigated using chi-square and Fisher’s exact tests. The spatial distribution of positive samples was plotted using the map tools package version 1.1 in R statistical software. A univariate Bernoulli scan statistic (BSS) model, implemented in SatScanTM version 10.2, was used to detect retrospective space–time significant clustering events [19]. Locations corresponding to positive samples were treated as cases, and those corresponding to negative samples were treated as controls. The scan statistic test null hypothesis assumes that the disease is randomly distributed in space and time. In contrast, the alternative hypothesis assumes that the disease in clustered in space and time. A significant scan statistic test suggests that the disease is clustered in space and time (i.e., the disease is dependent on the location where it was sampled and the time in the form of seasonality). Statistical significance was considered at *p* ≤ 0.05.

The model was set to scan for areas with high infection rates over a period of 7 days, and the maximum spatial window was configured to 10 km. *p*-values were obtained using 1000 Monte Carlo simulations.

## 3. Results

### 3.1. Animal Populations Management and Nomadic Nature

The sampled domestic animals were from 104 groups, each defined as a group of animals, either of a single- or multi-species, that grazed together within ~30 km^2^ and were owned by the same individual. The number of animals per group ranged from 31 to 80. Detailed information about animal movements was provided by only 22 group owners (21%). More than half of these owners (12/22; 54%) relocated their animals over long distances, ranging from 100 to 500 km from their original locations. The remaining owners (10/22) moved their animals within or near AlUla County, covering distances of less than 75 km from their original locations.

Animal husbandry practices varied across the target region. Most animal groups grazed in defined areas (63/104; 60%), while some grazed in pens (9/104; 8%) or on farms (24/104; 23%), and a few were free-grazing (3/104; 2.8%). No definite information was available for the remaining groups (5/104; 4.8%), but these were likely free-grazing. Regarding vaccination, most sampled groups (84/104; 81%) did not receive any vaccinations. Among vaccinated groups (16/104; 15%), six groups (37%) received both enterotoxemia and hemorrhagic septicemia vaccines, five groups (31%) received only the hemorrhagic septicemia vaccine, and two groups (12%) received other vaccines: one group was given only the enterotoxemia vaccine, and the other received both the camelpox and cowpox vaccines. The remaining two groups (12%) received an unspecified vaccine, as breeders were uncertain of the type. Vaccination status information was not provided by the owners of four groups (25%).

### 3.2. Prioritization of Infectious Diseases

The initial assessment identified 61 infectious diseases with potential for transmission from domestic and synanthropic animals to target wildlife species, including Arabian oryx and Nubian ibex, as well as mountain and sand gazelles, which are being reintroduced in the target region. Using the predefined scoring system, 11 of these diseases met the threshold (≥22 points) for high importance and were selected for field investigation. Of these, seven were bacterial, two were viral, and two were parasitic diseases. Brucellosis received the highest priority score (27), followed by FMD (23) and PPR (22). An additional nine diseases were classified as moderately important (scoring 17–21 points). These diseases were not included in the current round of field screening, but remain relevant for future monitoring and surveillance. Final scores for each disease are summarized in Supplementary Table S2.

### 3.3. Prevalence of Infectious Diseases

**Figure 2:**
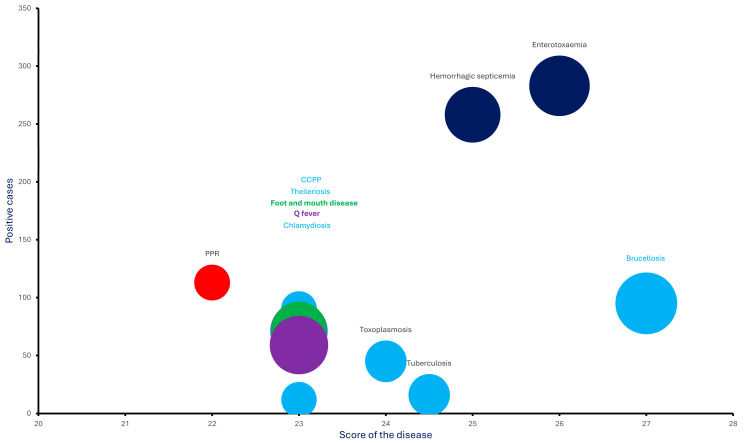
Scatter plot illustrating the distribution of positive disease cases. The *x*-axis represents the prioritization score assigned to each disease, the *y*-axis shows the number of positive cases detected, and the bubble size indicates the total number of samples tested for each respective disease. Representative colors displaying different diseases, i.e., Red: PPR; Blue: CCPP, Theileriosis, Chlamydiosis, Toxoplasmosis, Tuberculosis, Brucellosis; Dark Blue: HS, Enterotoxaemia; Green, FMD; Purple: Q fever.

Overall, 11 diseases were prioritized based on scoring criteria considering multiple epidemiological factors, which displayed significant correlations (Figure 2). Six disease-causing agents were detected by qPCR, with hemorrhagic septicemia (29.4%; 95% CI: 0.8–68.6%) and enterotoxemia (27.4%; 95% CI: 0–97%) being most prevalent, followed by theileriosis (7.6%; 95% CI: 0–44.4%), chlamydiosis (4.9%; 95% CI: 0–6.7%), Q fever (3.7%; 95% CI: 0–7.8%), and brucellosis (2.1%; 95% CI: 0–5.5%). Five diseases (PPR, FMD, CCPP, tuberculosis, and toxoplasmosis) were not detected by qPCR, but were identified serologically. Among these, PPR (31%; 95% CI: 0–54%) showed the highest seroprevalence, followed by Q fever (26.6%; 95% CI: 4.3–59%), FMD (25%; 95% CI: 24.7–25.7%), and brucellosis (17.8%; 95% CI: 2.7–22.6%). Serology also confirmed exposure to toxoplasmosis (9.3%; 95% CI: 0–31.2%), chlamydiosis (8.1%; 95% CI: 1.1–21%), CCPP (3.4%; 95% CI: 0–10.3%), and tuberculosis (3.1%; 95% CI: 0–12.7%) (Supplementary Table S4).

**Figure 3:**
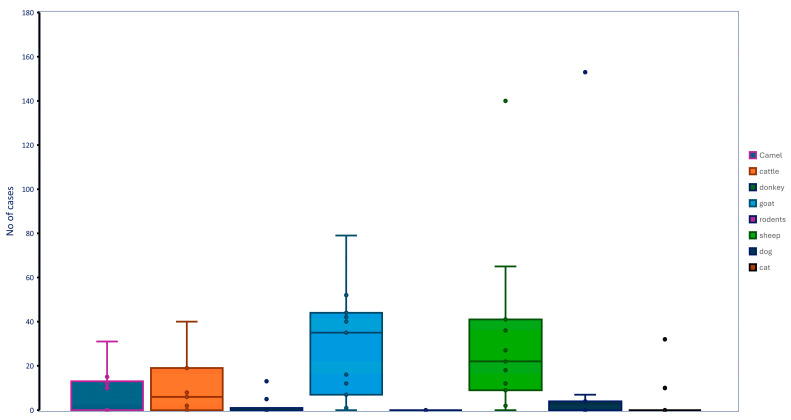
Box-Whisker plot presentation of positive cases of prioritized infectious diseases across different animal species. Each color represents a distinct animal species, e.g., camel: blue; cattle: brown; donkey: green; goat: blue; rodents: magenta; sheep: olive green; gray: dogs; mocha: cats, while dots within the boxes indicate individual data points. Outliers are marked as points beyond the whiskers.

Hemorrhagic septicemia was most frequently detected by qPCR in sheep, goats, camels, and cattle, with only isolated cases found in donkeys and rodents. Chlamydiosis was also detected in camels, goats, and sheep, while cattle showed serological evidence of exposure. Brucellosis was detected in goats and sheep, with serological evidence of exposure also found in cattle. Q fever was detected across all domestic ruminants (camels, cattle, goats, and sheep), but qPCR detection in camels and cattle was limited to single cases. Toxoplasmosis seroprevalence was highest in cats, followed by camels, dogs, cattle, donkeys, sheep, and goats. Serological evidence of tuberculosis exposure was observed only in goats and sheep (Table 2; Figure 3).

### 3.4. Determinants of Prioritized Diseases

The results reveal that chlamydiosis (5.46%, 95% CI: 0–6.7%), brucellosis (4.95%, 95% CI: 0–6.7%), and Q fever (4%, 95% CI: 0.7–7.8%) were found only in domestic animals, with no evidence of these diseases in synanthropic species. In contrast, enterotoxemia, PPR, toxoplasmosis, theileriosis, and hemorrhagic septicemia were found in both domestic and synanthropic animals. Hemorrhagic septicemia showed a significantly higher prevalence in domestic animals (*p* < 0.01), whereas enterotoxemia was more prevalent in synanthropic animals. Overall, domestic animals exhibited greater disease diversity and higher prevalence across most prioritized pathogens (Supplementary Table S4).

With respect to animal sex, chlamydiosis, brucellosis, and Q fever were detected by qPCR only in females. Hemorrhagic septicemia and theileriosis showed significantly higher prevalence in females compared to males (*p* < 0.01). Seasonal influence was also evident, with chlamydiosis, brucellosis, and Q fever being significantly more prevalent during winter months (*p* < 0.01). Enterotoxemia (*p* = 0.04) and hemorrhagic septicemia were detected in autumn and spring as well, but their prevalence was highest in winter. ELISA results show that CCPP, brucellosis, FMD, and tuberculosis had lower prevalence in autumn and spring. Furthermore, animals raised in defined grazing areas were more likely to harbor various diseases, with hemorrhagic septicemia being significantly more common (*p* < 0.01) (Table 3). Animals raised on farms also showed susceptibility to different diseases, but to a lesser extent compared to those raised in defined grazing areas.

### 3.5. Spatial Distribution of Prioritized Diseases

**Figure 4:**
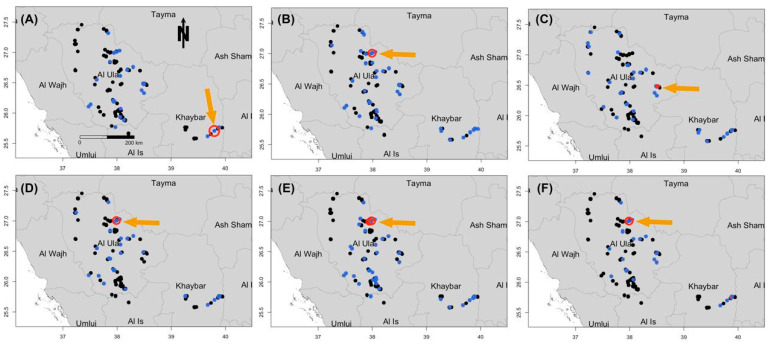
Spatial distribution of the most prevalent infectious diseases in the study area. Each panel (**A**–**F**) shows the location of sampled animals (black dots) and positive cases (blue dots). Red circles highlight significant spatial clusters identified using Bernoulli’s space–time scan statistics. (**A**) Peste des Petits Ruminants (PPR), (**B**) Hemorrhagic Septicemia, (**C**) Enterotoxemia, (**D**) Foot and Mouth Disease (FMD), (**E**) Q Fever, and (**F**) Brucellosis.

The findings show that hemorrhagic septicemia, chlamydiosis, enterotoxemia, and brucellosis were significantly prevalent (*p* < 0.01) across most reserves, especially in Harrat Khaybar, Al-Ghrameel, and Wadi Nakhlah. Theileriosis was mostly found near AlUla city, along with a few cases in Harrat Khaybar and Wadi Nakhlah reserves. Enterotoxemia was widespread across all reserves, with the highest prevalence recorded in Wadi Nakhlah, followed by Harrat Khaybar and Harrat Uwayrid, showing significant spatial variation (*p* < 0.01). Brucellosis cases were distributed throughout all reserves. Serological analysis showed the presence of PPR, chlamydiosis, brucellosis, FMD, Q fever, and tuberculosis in most reserves, with FMD identified in all reserves (Figure 4).

### 3.6. Univariate Space–Time Scan-Significant Clusters

Clusters for enterotoxemia were identified along the northern borders of the Wadi Nakhlah and Harrat Khaybar reserves. Hemorrhagic septicemia clusters were predominantly found in the southwestern region of Al-Ghrameel, the northwestern portion of Wadi Nakhlah, and the northern area of Harrat Khaybar. Similarly, clusters of brucellosis were detected in the southwestern part of Al-Ghrameel, central-western Wadi Nakhlah, the northeastern section of Harrat Al-Zabin, and the northern region of Harrat Khaybar. Clusters for Q fever were identified along the western border between Al-Ghrameel and adjacent to the Sharaan reserve boundary. FMD clusters were located along the northern border of Harrat Khaybar, southern Al-Ghrameel, the southwestern area adjacent to Sharaan, and the northeastern and northwestern parts of Harrat Al-Zabin (Table 2). PPR clusters were located along the western border between Al-Ghrameel and adjacent to Sharaan, the western region of Wadi Nakhlah, and the northwestern borders of Harrat Al-Zabin and Harrat Khaybar. Chlamydiosis clusters were detected near the northern border adjacent to Sharaan, the eastern part of Harrat Al-Zabin, the desert between Wadi Nakhlah, and the northern portion of Harrat Uwayrid. Toxoplasmosis clusters were identified in southern Al-Ghrameel and the northern region adjacent to Sharaan. Tuberculosis clusters were observed in the western area adjacent to Sharaan, northwestern Wadi Nakhlah, and the northern border of Harrat Khaybar. Overall, these results suggest that there are strong space–time interactions for all selected diseases, indicating that they are dependent on the location from which they were sampled (i.e., high-risk areas) and the time period in the form of temporal duration (i.e., their prevalence has a significant relationship with seasonality).

## 4. Discussion

The economic and ecological impacts associated with infectious diseases spreading from domestic and synanthropic animals to wild animals have become a significant global concern. Recent studies indicate an increase in emerging and re-emerging cases of viral, bacterial, and parasitic diseases throughout the Arabian Peninsula, including Saudi Arabia. Historical analyses within Saudi Arabia have documented the presence of various infectious diseases, including hemorrhagic septicemia, chlamydiosis, Q fever, brucellosis, tuberculosis, enterotoxemia, CCPP, FMD, PPR, theileriosis, and toxoplasmosis [20]. These diseases threaten wildlife populations, pose a zoonotic risk to human health, and critically impact the productivity and welfare of domestic animals. Therefore, this study aimed to investigate and assess the risk of infectious diseases that threaten reintroduced wildlife by screening domestic and synanthropic animals within conservation reserves in AlUla and Khaybar, Saudi Arabia.

Given the encroachment of human activities on natural habitats in AlUla, substantial efforts have been made to restore ecological integrity, including the reintroduction of highly threatened wildlife species such as gazelles, Arabian ibex, and Nubian oryx. As an essential step, a comprehensive analysis of the target ecosystem was conducted to evaluate the transmission risk of infectious diseases to these reintroduced species. The expert review resulted in 61 diseases potentially present within the study area. Through a structured multi criteria decision analysis (Figure 3), a total of 11 infectious diseases were prioritized for investigation. This decision analysis approach is well-established and widely employed in contemporary epidemiological research. Following recommendations by the European Centre for Disease Prevention and Control (ECDC), expert opinion was used as an essential source of evidence, particularly in scenarios with limited empirical data [21]. To minimize potential analytical bias, the Technical Working Group was purposefully structured to clearly reflect the objectives and scope of the prioritization process.

This study includes diverse animal populations across an extensive geographic area, thereby capturing representative population dynamics. Domesticated and synanthropic animals remain integral to AlUla’s ecological landscape. Synanthropic animals may act as critical bridge hosts facilitating disease transmission between domestic and wildlife populations [22]. Prior research has underscored the importance of biodiversity in influencing disease emergence, emphasizing the global role of domesticated and synanthropic animals as potential sources for such infections [23]. For instance, wild mammals were identified as primary disease hosts in Southeast Asia, with synanthropic species being approximately 15 times more likely to transmit pathogens compared to other wildlife hosts, particularly in regions experiencing habitat conversion and biodiversity loss [24].

In alignment with these global observations, our findings highlight the potentially significant role of synanthropic animals as sources of infectious pathogens in AlUla. Specifically, we observed a notably high prevalence of enterotoxemia among synanthropic dogs and cats, underscoring their potential contribution to disease spread in the region. The ecological characteristics of AlUla, including resource availability and interactions between domestic and synanthropic animals, provide favorable conditions for the emergence and transmission of infectious diseases. Shared food and water resources may serve as critical hubs for disease transmission. A recent ecological assessment in AlUla similarly documented feral donkeys’ potential role in contaminating water sources, thereby increasing disease transmission risks to wild animals [4]. Furthermore, these synanthropic populations negatively impact native plant communities, potentially undermining conservation and restoration efforts. Our findings are in parallel with previous studies from Saudi Arabia that report a significant enterotoxemia seroprevalence (27%) among synanthropic animals [25]. In addition, we observed a significantly higher seroprevalence of toxoplasmosis in synanthropic dogs and cats, similar to those reported in the Hail and Tabuk regions of Saudi Arabia [26].

Findings from the current study reveal that PPR was among the most prevalent disease among the eleven diseases tested in AlUla and Khabar provinces. High small-ruminant densities and the presence of local livestock markets, frequently supplied through animal imports via Jeddah, likely contribute to this elevated PPR seroprevalence. Similar conditions have been observed in other regions of Saudi Arabia, particularly near the southern borders adjacent to Yemen, a known PPR-endemic country [27,28]. In addition, the high prevalence of hemorrhagic septicemia (29.4%) represents a significant threat to wildlife. The WOAH acknowledges hemorrhagic septicemia as a major cause of livestock mortality across the Arabian Peninsula, Africa, and Asia. Moreover, brucellosis, FMD, and Q fever all demonstrated significant levels of prevalence or seroprevalence (*p* ≤ 0.01; Table 2). Q fever showed a seroprevalence of 26.6% in our study, similar to seropositivity rates reported recently in Saudi Arabia [29,30].

In this study, different rick factors affecting the prevalence of diseases were also identified. Spatial distribution significantly varied across different study locations. Seasonal variations, husbandry practices, and grazing patterns were significantly associated with the overall prevalence and distribution of infectious diseases, except in cases of toxoplasmosis and tuberculosis. These associations align with previous observations emphasizing the role of local animal densities, hygienic conditions, and grazing practices in shaping disease dynamics, as also reported in a study from Makkah Province [30]. In addition, significant space–time clusters were identified for several diseases, spanning substantial areas within the region. Space–time cluster analysis is instrumental in understanding disease spread and temporal dynamics within targeted populations and geographic regions. This analytical method is widely recognized for its effectiveness, as demonstrated in the epidemiological surveillance of various infectious diseases, including COVID-19 at the county level in the United States [31,32]. Its broad acceptance likely stems from its ability to integrate multiple epidemiological variables concurrently [33].

While this study provides a comprehensive epidemiological assessment of infectious diseases in AlUla through extensive sampling, it is limited by the absence of advanced genomic analyses such as whole-genome sequencing and functional genomics. Future investigations incorporating these advanced techniques could generate more comprehensive datasets, facilitating more targeted and effective preventive and control measures.

Building on these findings, subsequent research should broaden molecular screening to include additional diseases identified during the expert review and disease prioritization process. Although this study targeted diseases classified as highly important, including diseases of moderate priority would further strengthen the epidemiological baseline (Supplementary Table S2). These species have demonstrated the capacity to carry and transmit disease-causing agents, often via shared environmental resources such as water and grazing sites. Their proximity to both domestic and wild animals create a critical interface for disease spillover, which warrants proactive mitigation strategies in the target region. In parallel, domestic animal health management should be strengthened. The limited vaccination coverage observed among livestock in AlUla suggests an urgent need for systematic vaccination campaigns targeting priority diseases. Public awareness efforts and collaboration with livestock owners, breeders, and field personnel are also important in any future control program to ensure sustained participation and impact.

Another key limitation of the present study is that we could not quantify confounding relationships using multivariate statistical analyses such as logistic or multinomial regression models. Although we found statistically significant relationships with risk factors such as sex and animal species (Table 2 and Table 3), fitting a traditional statistical model is inappropriate because of a strong space–time interaction that shapes the risk of our disease outcomes. Therefore, the presence of significantly strong spatiotemporal structure in our data requires specialized analysis methods, such as Bayesian hierarchical spatiotemporal models. However, conducting such complex analysis requires focusing on one or two disease outcomes, which is beyond the scope of this study.

Wildlife health surveillance should be enhanced through an integrated monitoring plan aligned with conservation objectives. Particular attention should be directed toward highly endangered species, including those targeted for future reintroduction such as the Arabian leopard (*Panthera pardus nimir*). Early detection of pathogen risks in prey populations and environmental reservoirs will be essential to support safe and sustainable reintroduction programs. It is also recommended to incorporate target-independent diagnostic tools, such as metagenomic sequencing, to help identify emerging or previously undetected pathogens and support the development of early warning systems. Such approaches, coupled with assessments of pathogen inflow from environmental sources, including surface water and migratory birds, will strengthen biosecurity and inform regional One Health strategies. A broader integration of wildlife disease monitoring into national health frameworks is also advised to address potential risks at the human–animal–environment interface.

## 5. Conclusions

This study presents one of the first epidemiological assessments targeting infectious diseases at the domestic, synanthropic, and wild animal interface within AlUla’s conservation reserves, which are being targeted for the reintroduction of highly threatened wildlife species. By prioritizing 11 high-impact diseases and conducting large-scale molecular and serological screening, we identified clear evidence of pathogen presence in domestic and synanthropic species, highlighting the risk of spillover to reintroduced or native wildlife. These findings provide insight into disease dynamics across species and landscapes and offer critical guidance for the development of control strategies. Importantly, this study goes beyond passive surveillance by proactively assessing pathogen risks in advance of reintroduction efforts, an approach that strengthens wildlife survival and supports the long-term success of conservation programs. The recommendations derived from our findings serve as a foundation for targeted prevention, vaccination, and biosecurity measures. Taken together, this study delivers a model for integrating health surveillance into reintroduction planning and contributes to the broader One Health framework essential for sustaining biodiversity in arid and semi-arid ecosystems.

## Figures and Tables

**Table 1:** Details of primer and probe sequences used for pathogen detection in this study.

Disease	Primer and Probe Sequences	Annealing Temperature (°C)	qPCR Confirmation	Reference
Brucellosis (*Brucella* spp.)	F: GCTCGGTTGCCAATATCAATGC R: GGGTAAAGCGTCGCCAGAAG P: FAM-AAATCTTCCACCTTGCCCTTGCCATCA-TAMRA	60	Yes	[10]
Enterotoxemia (*Clostridium perfringens*)	F: AAGAACTAGTAGCTTACATATCAACTAGTGGTG R: TTTCCTGGGTTGTC-CATTTCC P: VIC-TTGGAATCAAAACAAAGGATGGAAAAACTCAAG-TAMRA	54	Yes	[11]
Hemorrhagic septicemia (*Pasteurella multocida*)	F: CAGAGTTTGGTGTGTTGA R: CAGACTGACAAGGAAATATAAAC P: FAM-AATCTGCTTCCTTGAC-BHQ1	52	Yes	[12]
Tuberculosis (*Mycobacterium bovis*)	F: GGCTGCTCTCGACGTTCATC R: CGCTGATTGGACCGCTCAT P: FAM5′-CTGAAGCCGACGCCCTGTGC-BHQ	55	No	[13]
Contagious Caprine Pleuropneumonia (*Mycoplasma capricolum* subsp. Capripneumoniae)	F: TTTTTCAAGTGCAAACGACTATG R: TGACTTGGGTGTTAGGACCA P: Cy5-CGGATAGAACAATAGCTTTTACAGA-BHQ	52	No	[11]
Q fever (*Coxiella burnetii*)	F: CGTTATTTTACGGGTGTGCCA R: CAGAATTTTCGCGGAAAATCA P: FAM-CATATTCACCTTTTCAGGCGTTTTGACCGT-TAMRA	52	Yes	[14]
Chlamydiosis (Chlamydia psittaci)	F: CACTATGTGGGAAGGTGCTTCA R: CTGCGCGGATGCTAATGG P: FAM-CGCTACTTGGTGTGAC–TAMRA ^1^	50	Yes	[15]
Chlamydiosis (Chlamydia Abortus)	F: GCAACTGACACTAAGTCGGCTACA R: ACAAGCATGTTCAATCGATAAGAGA P: FAM-TAAATACCACGAATGGCAAGTTGGTTTAGCG–TAMRA ^1^	50	Yes	[15]
Peste des petits ruminants	F: CCATCAYTACCCGTTCAAG R: ATYCGCTGKATCARTTGC P: HEX-GIGACTCYACGAACA-BHQ1	47	No	[12]
Foot and Mouth Disease	F: ACTGGGTTTTACAAACCTGTGA R: GCGAGTCCTGCCACGGA P: FAM-TCCTTTGCACGCCGTGGGAC-TAMRA	60	No	[16]
Toxoplasmosis (*Toxoplasma gondii*)	F: TCCCCTCTGCTGGCGAAAAGT R: AGCGTTCGTGGTCAACTATCGATTG P: FAM-TCTGTGCAACTTTGGTGTATTCGCAG-TAMRA	50	Yes	[17]
Theileriosis (Theileria annulata)	F: AGACCTTAACCTGCTAAATAGG R: CATCACAGACCTGTTATTGC P: FAM-AAGTTTCTACTGTCCCGTT-BHQ ^2^	50	Yes	[18]
Theileriosis (Theileria orientalis)	F: GGAAACCAAGGATCTCGATG R: GAATGGTCCGACGAAGTCAT P: JOE-TTGCAGAGGCAGGTCTTTTT-BHQ ^2^	50	Yes	[18]

^1^ qPCR uniplex; ^2^ qPCR multiplex.

**Table 2:** Prevalence and seroprevalence of prioritized infectious diseases across animal species.

Animal Diseases and Number of Samples Investigated		qPCR									
		Number of Animals Detected Positive									
Diseases	Processed Samples	Camels N = 254	Cattle N = 90	Donkey N = 111	Goat N = 290	Rodents N = 123	Sheep N = 294	Dog N = 168	Cat N = 37	Overall % Prevalence	*p*-Value
Theileriosis	934	0 ^1^	40	0	0	0	27	4	0	7.59	≤0.01
Enterotoxemia	1032	15	8	13	40	0	22	153	32	27.42	≤0.01
Hemorrhagic septicemia	876	31	6	1	79	1	140	– ^5^	–	29.45	≤0.01
Chlamydiosis	609	8	0	0	11	0	11	0	0	4.9	0.119
Brucellosis	605	0	0	0	9	0	4	0	–	2.11	0.06
Q fever	656	1	1	0	15	0	7	0	0	3.66	0.016
ELISA											
PPR ^2^	364	0	–	–	44	–	65	4	–	31.04	≤0.01
Chlamydiosis	360	4	19	–	5	–	1	–	–	8.06	≤0.01
CCPP ^3^	352	0	–	–	12	–	0	–	–	3.41	≤0.01
Brucellosis	460	–	2	–	43	–	37	–	–	17.83	≤0.01
FMD ^4^	360	–	19	–	35	–	36	–	–	25.00	0.99
Q fever	184	9	2	–	27	–	11	–	–	26.63	≤0.01
Toxoplasmosis	484	13	7	5	1	0	2	7	10	9.30	0.003
Tuberculosis	484	0	0	0	7	0	9	0	0	3.31	≤0.01

^1^ Number of positive cases; ^2^ PPR, peste des petits ruminants; ^3^ CCPP, contagious caprine pleuropneumonia; ^4^ FMD, foot and mouth disease. ^5^ A dash (–) indicates that ELISA testing was not performed for that species because the disease is either not known to affect the species, lacks historical reports in that host, or no validated ELISA exists for that disease.

**Table 3:** Distribution of prioritized infectious diseases by sex and animal husbandry practices.

Diseases	Sex			Husbandry					
	qPCR								
	Female	Male	* p * Value	Farms	Free Grazing	Grazing in Defined Areas	In Pen	ND *	* p * Value
Theileriosis	61	10	0.327	40	0	21	3	7	≤0.01
Enterotoxemia	188	95	0.030	10	0	52	18	203	0.44
Hemorrhagic septicemia	239	19	<0.01	24	0	220	11	3	≤0.01
Chlamydiosis	30	0	1	0	0	30	0	0	0.001
Brucellosis	13	-	1	1	0	12	0	0	0.59
Q fever	24	0	1	1	0	23	0	0	0.06
	ELISA								
PPR ^1^	98	15	0.499	5	0	88	8	12	0.93
Chlamydiosis	27	2	0.281	22	0	6	0	1	≤0.01
CCPP ^2^	12	0	0.225	0	0	12	0	0	0.68
Brucellosis	74	8	1	6	0	61	0	15	≤0.01
FMD ^3^	86	4	0.029	23	0	58	9	0	0.07
Q fever	42	7	0.186	5	0	37	3	4	≤0.01
Toxoplasmosis	34	11	0.822	7	0	15	6	17	0.62
Tuberculosis	13	3	0.532	1	0	11	2	2	0.14

^1^ PPR, peste des petits ruminants; ^2^ CCPP, contagious caprine pleuropneumonia; ^3^ FMD, foot and mouth disease; *p*-values were estimated using Fisher’s exact chi-square test. * Animals in this category could not be assigned to any of the defined husbandry practices due to insufficient details provided during sample collection.

## Data Availability

The data supporting the findings of this study are not publicly available due to institutional and legal restrictions but may be provided by the corresponding author upon request and subject to approval from the Royal Commission for AlUla.

## Supplementary Materials

The following supporting information can be downloaded at: https://www.mdpi.com/article/10.3390/vetsci12090836/s1, Supplementary Table S1: criteria and scoring system used for expert-based prioritization of diseases, Table S2: final scores for infectious diseases based on expert evaluation using the defined criteria, Table S3: sample sizes for each animal species in each reserve, and Table S4: summary of qPCR and ELISA results for each disease by animal species.
