# Supplementary material for: Epidemiological Investigation of Infectious Diseases at the Domestic–Synanthropic–Wild Animal Interface Reveals Threats to Endangered Species Reintroduction in AlUla, Saudi Arabia
Source: Vet Sci. 2025 Aug 30;12(9):836. doi: 10.3390/vetsci12090836 (PMC12474512; doi:10.3390/vetsci12090836)
Supplement: Supplementary file 1 [file vetsci-12-00836-s001.zip › vetsci-3778177-supplementary-new.pdf]

Supplementary Table S1. Criteria and scoring system used for expert-based prioritization of diseases.

Query	Response	Score
Vector-borne transmission	Yes	1
	No	3
Susceptible domestic Reservoirs (Camels, sheep, goats, cattle)	Camels	3
	Sheep/goat	3
	Cattle/other animals	1
	More than one domestic animal	4
Susceptible Wildlife hosts	Ibex, Oryx, or Gazelles	3
	Two species from the above	6
	Potential to infect Ibex, Oryx, or Gazelles	2
	Other wildlife	1
Susceptible synanthropic animals (donkeys, dogs, cats, and rodents)	One species	1
	Two species	2
Likelihood of occurrence[1]	Very likely	6
	Likely	4.5
	Possible	1.5
	Minor possibility or remote	1
Severity on Wildlife[2]	Catastrophic	6
	Critical	5
	Moderate	3
	Minor	1
Expert opinion	Variable	(+/-2)

[1]Likelihood of occurrence is based on documented or potential disease presence in AlUla, Saudi Arabia, or nearby regions, ranging from very likely (recently documented locally) to remote (not detected for decades).

[2]Severity on wildlife reflects potential impact on wild species, especially Ibex, Oryx, and Gazelles, ranging from catastrophic (severe population decline) to minor (mild or asymptomatic illness).

Supplementary Table S2. Final scores for infectious diseases based on expert evaluation using the defined criteria.

Disease Rank		Name of the Disease	Score
**High Importance**	1	Brucellosis	27
	2	Enterotoxaemia	26
	3	Hemorrhagic septicemia	25
	4	Tuberculosis	24.5
	5	Toxoplasmosis	24
	6	Foot and mouth disease	23
	7	Theileriosis	23
	8	Contagious Caprine	23
	9	Q fever	23
	10	Chlamydiosis	23
	11	Peste des petits ruminants	22
**Moderate importance**	12	Salmonellosis	21
	13	Neosporosis	21
	14	Listeriosis	20.5
	15	Bluetongue	20
	16	Ectoparasitism*	20
	17	Paratuberculosis	20
	18	West Nile Fever	18.5
	19	Sarcocystosis	18.5
	20	Echinococcosis	17.5
	21	Babesiosis	17
	22	Anaplasmosis	17

*Ectoparasitism refers to infestations by external parasites such as ixodid ticks (vectors for various diseases) and mites (causative agents of mange in livestock).

Supplementary Table S3. Sample sizes for each animal species in each reserve.

Animal Species	Harrat Al-Zabin	Wadi Nakhlah	Wadi Nakhlah & Harrat Al-Zabin[1]	Harrat Khaybar	Sheraan	Al-Ghrameel	Harrat Uwayred	AlUla County[2]
Camels	25	65	46	22	-	34	62	-
Sheep	50	85	29	34	3	33	60	-
Goats	50	79	27	34	1	33	66	-
Donkeys	-	-	-	1	-	-	-	110
Mice	-	-	-	1	-	-	-	34
Gerbil	-	-	-	24	-	-	-	64
Cattle	-	-	-	14	-	-	-	76

[1]Animals located outside the boundaries of Wadi Nakhlah and Harrat AlZabin, but situated between them; [2]Animals sampled within the county but not in proximity to any specific reserve

Supplementary Table S4. Summary of qPCR and ELISA results for each disease by animal species

Disease	Test	Camels		Cattle		Sheep		Goat		Donkey		Dogs		Cats		Rodents		P-Value
		+/n[1]	%	+/n	%	+/n	%	+/n	%	+/n	%	+/n	%	+/n	%	+/n	%	
Theileriosis	PCR	0/ 162	0	40/90	44.4	27/ 203	13.3	0/ 194	0	0/ 107	0	4/ 91	4.4	0/ 30	0	0/ 57	0	< 0.01*
Enterotoxaemia		15/161	9.3	8/ 78	10.3	22/ 200	11	40/192	21	13/97	13.4	153/161	95	32/33	97	0/ 110	0	< 0.01*
Hemorrhagic septicemia		31/163	19	6/ 90	6.7	140/ 204	68.6	79/194	40.7	1/ 104	0.96	-[2]	-	-	-	1/ 121	0.8	< 0.01*
Chlamydiosis		8/ 140	5.7	0/ 75	0	11/ 170	6.5	11/164	6.7	0/ 2	0	0/ 24	0	0/ 3	0	0/ 31	0	0.119
Brucellosis		0/ 141	0	0/ 75	0	4/ 170	2.4	9/ 165	5.5	0/ 2	0	0/ 23	0	-	-	0/ 29	0	0.064
Q fever		1/ 141	0.7	1/ 75	1.3	7/ 188	3.7	15/192	7.8	0/20	0	0/ 240	0	0/ 30	0	0/ 31	0	0.016*
PPR	ELISA	0/ 92	0	-	-	65/ 121	54	44/123	36	-	-	4/ 28	14	-	-	-	-	< 0.01*
Chlamydiosis		4/ 91	4.4	19/89	21	1/ 90	1.1	5/ 90	5.6	-	-	-	-	-	-	-	-	< 0.01*
CCPP		0/ 120	0	-	-	0/ 115	0	12/117	10.3	-	-	-	-	-	-	-	-	< 0.01*
Brucellosis		-	-	2/ 74	2.7	37/ 196	19	43/190	22.6	-	-	-	-	-	-	-	-	< 0.01*
FMD		-	-	19/74	25.7	36/ 146	24.7	35/140	25	-	-	-	-	-	-	-	-	0.987
Q fever		9/ 46	19.6	2/ 46	4.3	11/ 46	23.9	27/46	59	-	-	-	-	-	-	-	-	< 0.01*
Toxoplasmosis		13/84	15.5	7/ 62	11.3	2/ 71	2.8	1/ 68	1.5	5/ 90	5.6	7/ 50	5.6	10/32	31.2	0/ 27	0	0.003*
Tuberculosis		0/ 84	0	0/ 61	0	9/ 71	12.7	7/ 68	10.3	0/ 90	0	0/ 50	0	0/ 33	0	0/ 27	0	< 0.01*

[1]Number of positive cases over the total number of samples tested by qPCR or ELISA.

[2]A dash (–) indicates that ELISA testing was not performed for that species because the disease is either not known to affect the species, lacks historical reports in that host, or no validated ELISA assay exists for that disease.
